# Update of the pediatric hypotension graphic adjusted for gender and height percentiles: systolic blood pressure for girls, 1 to 17 years old

**DOI:** 10.1186/cc12638

**Published:** 2013-06-19

**Authors:** HH Shieh, ER Barreira, A Bousso, AC Ventura, EJ Troster

**Affiliations:** 1 University Hospital of Universidade de São Paulo, Vila Iara, São Paulo, SP, Brazil

## Introduction

According to the National Heart, Lung, and Blood Institute of the National Institute of Health, hypotension refers to an abnormally low blood pressure (BP). In childhood, hypotension can be determined according to two different definitions: BP below the 5th percentile or below two standard deviations (SDs) of the mean for age and gender [1]. A graphic representation of pediatric hypotension was published in 1977 [2], and no updates have been published since then. The objective of this study was to update the graphic representation of pediatric hypotension.

## Methods

We used a computerized calculation method to develop high-resolution graphics containing curves with 5,841 points each, to depict the main percentiles associated with low BP for girls from 1 to 17 years old in the 50th percentile of height. Each point represents the calculation of the polynomial equation that includes the statistical processing of the last Report on Blood Pressure in 2004 [3]. We also analyzed the effect of height on BP from the 5th to 95th percentile. Statistical functions generated by computerized program were used.

## Results

**Figure 1:**
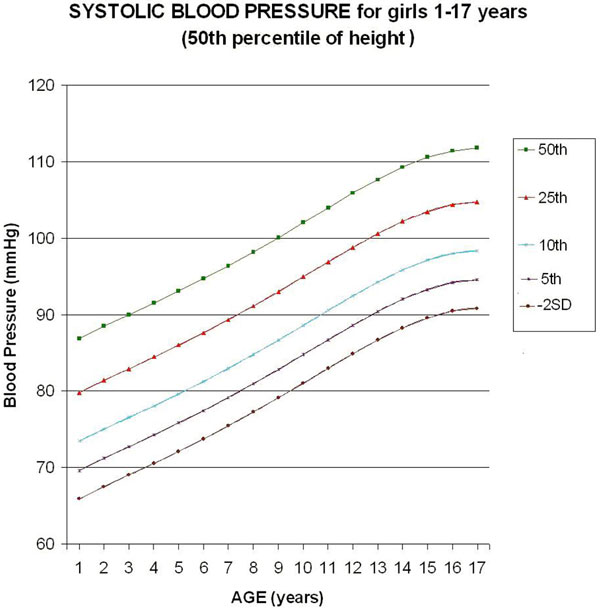
**Update of chart for systolic blood pressure (SBP) based on the last Report on Blood Pressure in 2004 **[3]**, for girls 1 to 17 years old (50th percentile of height)**. Considering a tolerance of 1 mmHg, the curve of adjustment for height of the SBP for girls does not need any correction in the in the 31.5th to 68.5th percentile of height.

Five monotonic curves of systolic BP for girls representing the 50th, 25th, 10th, 5th, and 2.275th (-2SD) percentiles were built (Figure 1). In relation to the table published by Haque and Zaritzsky [3], the Bland-Altman analysis of the published female systolic BP compared with actual update shows a mean bias of -0.61, with limits of agreement from -2.12 to 0.9 mmHg, which confirms the use of approximation of the values in the published table [3]. Considering a tolerance of 1 mmHg, the monotonic curve of adjustment for height of the SBP for girls does not need any correction in the 31.5th to 68.5th percentile of height, but needs maximal correction for the 5th percentile for height (-4.7 mmHg correction).

## Conclusion

The correction of systolic BP for height is of little significance, and this updated graphic can be used to diagnose low systolic BP for girls. Clinical studies are necessary to determine the systolic BP percentile that better represents clinically significant hypotension.
